# gga-miR-148a-5p-Targeting PDPK1 Inhibits Proliferation and Cell Cycle Progression of Avain Leukosis Virus Subgroup J (ALV-J)-Infected Cells

**DOI:** 10.3389/fcell.2020.587889

**Published:** 2020-12-15

**Authors:** Heling Yu, Hengyong Xu, Chaoyang Yan, Shiliang Zhu, Xi Lan, Yuxiang Lu, Qijian He, Huadong Yin, Qing Zhu, Xiaoling Zhao, Diyan Li, Yiping Liu, Yan Wang

**Affiliations:** ^1^Farm Animal Genetic Resources Exploration and Innovation Key Laboratory of Sichuan Province, Sichuan Agricultural University, Chengdu, China; ^2^College of Animal Science and Technology, Southwest University, Chongqing, China

**Keywords:** chicken, ALV-J infection, gga-miR-148a-5p, PDPK1, proliferation, cell cycle

## Abstract

Avian leukosis virus subgroup J disease (ALV-J) is a contagious and immunosuppressive avian disease caused by ALV-J virus. Although miRNA participate in various biological processes of tumors, little is known about the potential role of miRNA in ALV-J. Our previous miRNA and RNA sequencing data showed that the expression of gga-miR-148a-5p was significantly different in ALV-J-infected chicken spleens compared with non-infected chickens. The aim of this study was to investigate the functional roles of gga-miR-148a-5p and identify downstream targets regulated by gga-miR-148a-5p in ALV-J-infected chickens. We found that the expression of gga-miR-148a-5p was significantly downregulated during ALV-J infection of chicken embryo fibroblasts (CEF). Dual luciferase reporter assays demonstrated that PDPK1 is a direct target gene of gga-miR-148a-5p. *In vitro*, overexpression of gga-miR-148a-5p significantly promoted ALV-J-infected CEF cell proliferation, included cell cycle, whereas inhibition of gga-miR-148a-5p had an opposite effect. Inhibition of PDPK1 promoted the proliferation of ALV-J-infected cells but had no effect on the activity of NF-κB. Together, these results suggested that gga-miR-148a-5p targets PDPK1 to inhibit the proliferation and cell cycle of ALV-J-infected CEF cells. Our study provides a new understanding for the tumor mechanism of ALV-J infection.

## Introduction

Avian leukosis viruses (ALVs) is a virus belong to the genus *Alpharetrovirus* of the *Retroviridae* family that can cause a variety of tumors in chicken (Hayward et al., 1981). At present, chicken ALVs can be classified into 10 subgroups according to the different avian leukosis virus envelope proteins, virus interference patterns, and host cells, including exogenous (subgroups A, B, C, D, J, and K) and endogenous (subgroups E, F, G, and H) viruses (Payne et al., 1991). Among these subgroups, the J subgroup of ALV (ALV-J) is the most infective subgroup, which was first reported in commercial chickens in 1988 (Payne et al., 1992). ALV-J infection can cause myeloid leukemia, various tissue tumors, and severe immunosuppression (Stedman and Brown, 1999; Payne and Nair, 2012; Zeng et al., 2014). Due to the high horizontal genetic variation and vertical and horizontal transmission of ALV-J, there is no effective vaccine and treatment against ALV-J until now (Payne and Nair, 2012). Therefore, ALV-J infection results in serious economic loss in poultry production consisting of reduced weight gain and egg production, as well as a large number of chicken deaths (Fadly and Smith, 1999; Nakamura et al., 2000). Although, there are three theories including promoter insertion, enhancer activation, and viral oncogenes to explain the pathogenesis of ALV-J (Payne, 1998; Li et al., 2015), the microribonucleic acids (miRNA) involved in ALV-J and associated signaling pathways remain unclear.

MiRNA are small non-coding RNAs that consist of 19-23 nucleotides, which bind to the 3′-untranslated regions (3′-UTR) causing translation inhibition and/or corresponding transcript degradation (O'Reilly, 2016). In recent years, accumulating evidence indicates that miRNAs play important roles in various biological processes including cell proliferation, differentiation, cell death (Lpez-Camarillo, 2013), inflammation (Karthikeyan et al., 2016), cancer (Rupaimoole and Slack, 2017; Sandiford et al., 2018), and immune response (Naqvi et al., 2018). Meanwhile, miRNAs also play critical roles in poultry diseases, such as Marek's disease (Li X. et al., 2014), avian leucosis (Li Z. et al., 2017), avian influenza (Wang et al., 2009), and infection bursal disease (Fu et al., 2017). To date, several miRNAs related to ALV-J have been discovered. For example, gga-miR-375 is downregulated in ALV-J by inhibiting cell proliferation through YAP1 oncogene targeting (Li H. et al., 2014). MiRNA-23b promotes ALV-J replication by targeting interferon regulator 1 (IRF1) (Li et al., 2015). MiR-221 may play an important role in the growth of ALV-J-infected DF-1 cells (Ren et al., 2018). In addition, seven miRNAs (miR-205a, miR-21-5p, miR-383-5p, miR-203, miR-223, miR-148a-5p, and miR-21-3p) have been reported to may play a pivotal role in tumorigenesis after ALV-J infection (Lan et al., 2017).

The miR-148 family consists of miR-148a, miR-148b, and miR-152. Among them, miR-148a has drawn our attention because it is a 17–25-nucleotide-long highly conserved single-stranded non-coding RNA and plays important roles in different tumors including ovarian cancer, breast cancer, and renal cell carcinoma, either as an oncogene or as a tumor suppressor (Gong et al., 2016; Cao et al., 2017; Li Q. et al., 2017). Overexpression of has-miR-148a inhibits hypertrophic differentiation, thus it might be a potential disease-modifying compound in osteoarthritis (Vonk et al., 2014). In addition, miR-148a acts through the sonic hedgehog signaling pathway to induce hepatic stellate cell autophagy and apoptosis (Liu X. Y. et al., 2015). However, to date, the role of gga-miR-148a-5p in ALV-J infection and possible mechanism remains unclear. Our previously published RNA sequencing data showed that gga-miR-148a-5p was downregulated in ALV-J-infected chicken compared with non-infected chickens (Ren et al., 2018); therefore, we hypothesized that gga-miR-148a-5p plays a role in ALV-J infection.

The purposes of this study was to investigate whether or not gga-miR-148a-5p is involved in ALV-J infection and elucidate how it affects the proliferation and cycle of chicken embryo fibroblasts (CEF) infected with ALV-J, thus inducing tumor. We verified that the expression level of gga-miR-148a-5p was decreased in ALV-J-infected CEF cells. PDPK1 was demonstrated as a direct target gene of gga-miR-148a-5p. Meanwhile, *in vitro* investigations found that lowering the expression of gga-miR-148a-5p can promote the expression of PDPK1, thus inhibiting cell proliferation and cycle of ALV-J-infected CEF cells. Taken together, these results indicate that gga-miR-148a-5p can inhibit the proliferation and cycle of ALV-J-infected CEF cells by targeting PDPK1, indicating that gga-miR-148a-5p is a potential interfering target that can improve the host's infection of virus and tumor formation.

## Materials and Methods

### Animal Ethics Statement

Specific pathogen-free (SPF) white Leghorn chicken eggs were purchased from Boehringer Ingelheim Vital Biotechnology Co., Ltd (Beijing, China). Incubation of SPF eggs in an incubator occurred under direction of animal genetics and breeding at Sichuan Agricultural University, School of Animal Science and Technology. All animal experiment procedures and sample collection followed the rulers approved by the Institutional Animal Care and Use Committees of Sichuan Agricultural University (Protocol number 2014-18).

### Cells and Viruses

Primary CEF and the DF-1 cell line were cultured in DMEM (Gibco, Grand Island, NY, US), supplemented with 10% fetal bovine serum (FBS, Glibco) and 100 U/ml penicillin-streptomycin at 37°C with 5% CO_2_ and 95% humidity. The CEF were isolated from 9 embryonic age SPF chickens (Merial, Beijing, China), following removal of its head, limbs, internal organs, bones, and blood. The entire experimental procedure was performed in a UV-sterilized cell compartment to ensure a sterile, non-polluting environment when collecting primary cells. The ALV-J were kindly provide by Associate Professor Peng Zhao from the College of Animal Science and Veterinary Medicine, Shandong Agricultural University, China.

### Bioinformatic Analysis

The microRNA Target Prediction and Functional Study Database (miRDB; http://www.mirdb.org/miRDB/index.html) and the target gene prediction databases TargetScan7.1 (http://www.targetscan.org/) were used to predict target genes of the seven differentially expressed miRNAs screened in our previous experiment (Li Z. et al., 2017). The target genes were then enriched by defining the intersection through the DAVID biological information network for NF-κB signal pathway enrichment and through the Omicshare cloud platform (https://www.omicshare.com/) and GO analysis of the intersection target genes. Then, using miRbase (http://www.mirbase.org/) and TargetScan to query the complementary binding sites of gga-miR-148a-5p and target genes, it was predicted that *PDPK1* was the target gene of gga-miR-148a-5p.

### Construction of PDPK1 Wild-Type Dual Luciferase Reporter Gene Plasmid

The miRNA mimic and inhibitor of gga-miR-148a-5p and their negative controls were chemically synthesized by GenePharma (Shanghai, China). Combining the miRDB (http://www.mirdb.org/cgi-bin/target_detail.cgi?targetID=541541) and NCBI databases, found positions 42, 2455, 3731, 3907, and 4110 of the *PDPK1* 3′-UTR were complementary to gga-miR-148a-5p. Three PDPK1 gene 3′-UTR wild-type reporter plasmids were constructed, using the PGL3-CMV-LUC-MCS vector (supplied by Shanghai Jiman Biotechnology Co, Ltd). First, the linearized vector was obtained by double enzyme digestion, and then the PCR-amplified fragment was obtained by PCR amplification. A homologous base with 20 linearized vector ends was designed and synthesized as follows: gga-PDPK1-F: 5′-AGATCGCCGTGTGACTCGAGTAACAATCAGACATGCAGTCACCTTGC-3′, gga-PDPK1-R:5′-CCCCGACTCTAGCACGCGTCCTTGACACAAGATTTGAAACTGCC-3′, with the black underlined part of the sequence being the homologous arm sequence, the unlined part being the upstream primer sequence and the downstream primer sequence of PDPK1, and the XhoI and MluI cleavage sites are the positions where the homologous arms are added at both ends of the primer. XhoI and MluI double-cut PGL3-CMV-LUC-MCS empty vector and the double-digested PCR product were purified by 2% agarose gel electrophoresis, according to the HieffClone™ recombination reaction system.

### PDPK1-siRNA Synthesis and Transfection

Three small interfering RNAs (siRNAs) against PDPK1 (siRNA-503, siRNA-631, and siRNA-794) and the negative control (siRNA-NC) were chemically synthesized by GenePharma (Shanghai, China), with sequences provided in Table 1. siRNA (5 μg/well) was transfected into chicken CEF at 70–80% confluence by using X-treme GENE siRNA Transfection Reagent in a six-well-plate, and qRT-PCR was performed to analyze the knockdown efficiency of *PDPK1*.

**Table 1:** Primer sequence.

**Genes**	**Primers (5**′**-3**′**)**	**Size (bp)**
si-PDPK1(503)	Forward:GCGAGCUGCUAAAGUAUAUTT	21
	Reverse:AUAUACUUUAGCAGCUCGCTT	21
si-PDPK1(631)	Forward: CCAGAGAACAUCUUGCUAATT	21
	Reverse: UUAGCAAGAUGUUCUCUGGTT	21
si-PDPK1(794)	Forward: GCUCUGACCUCUGGGCUUUTT	21
	Reverse: AAAGCCCAGAGGUCAGAGCTT	21

### RNA Isolation and qRT-PCR Analysis

Total cellular RNA was extracted from the cultured CEF by using RNAiso Plus reagent (TaKaRa, Dalian, China) according to the manufacturer's instructions. RNA concentration and purity were measured by Nanodrop 2000 (Thermo Fisher Scientific, MA, USA). After the concentration and purity of the RNA were determined, a total of 1 μl RNA was converted into cDNA using the PrimeScript RT Reagent Kit (TaKaRa, Dalian, China). However, in order to obtain the cDNA of gga-miR-148a-5p, we used the miRNA first-strand cDNA synthesis (stem-loop method) kit (Sangon, Shanghai, China) to make cDNA of gga-miR-148a-5p and internal reference gene U6 step by step. The real-time PCR was performed on a LightCycler96 qPCR system (Roche, USA) using One Step SYBR® PrimeScript™ RT-PCR Kit (TaKaRa, Dalian, China). U6 was used as an internal reference of gga-miR-148a-5p, and GAPDH was the internal control for PDPK1 and ENV. Each amplification reaction (10 μl) contained 1 μl cDNA or miRNA cDNA, 2 μl primers, and 5 μl SYBR solution. The following cycling conditions were used: one cycle predenaturation at 95°C for 3 min and 40 cycles of denaturation at 95°C for 10 s and amplification at 65°C for 30 s. All reactions were conducted in triplicate. The primers used in this study are listed in Table 2. All primers were chemically synthesized by Sangon Biotech Co, Ltd (Shanghai, China). To quantify the PCR products, the relative expression level of each gene was calculated and normalized using the 2^−ΔΔCt^ algorithm (Gong et al., 2016).

**Table 2:** Primer sequence for real-time PCR.

**Genes**	**Primers (5**′**-3**′**)**	**Size (bp)**
PDPK1	Forward: TCCCAGAGACAAGTACCCCA	20
	Reverse: TCCACGGGGCCCATTTTTAG	20
ENV	Forward: AGAAAGACCCGGAGAAGAC	19
	Reverse: ACACGTTTCCTGGTTGTT	18
GAPDH	Forward: AGGACCAGGTTGTCTCCTGT	20
	Reverse: CCATCAAGTCCACAACACGG	20
gga-miR-148a-5p	Forward: GCGCGAAAGTTCTGTGACACT	21
	Reverse: AGTGCAGGGTCCGAGGTATT	20
	RT:GTCGTATCCAGTGCAGGGTCCGAGGTATTCGCACTGGATACGACAGTCTG	50
U6	Forward: TTCGGCAGCACATATACTAAAATTGGA	27
	Reverse: CGAATTTGCGTGTCATCCTTGC	22

### Dual Luciferase Reporter Assay

To confirm whether PDPK1 is the direct target gene of gga-miR-148a-5p, wild-type (WT) dual luciferase reporter genes with correctly identified sequences were cotransfected with gga-miR-148a-5p into HEK293 cells. The test groups were gga-miR-148a-5p mimics+PDPK1 WT1+pRL-TK (Renilla luciferase reporter vector, acted as an internal reference), gga-miR-148a-5p mimics+PDPK1 WT2+pRL-TK, gga-miR-148a-5p mimics+PDPK1 WT3+pRL-TK, gga-miR-148a-5p mimics+UTR NC (control of target gene 3′-UTR)+pRL-TK, miRNC+PDPK1 WT1+pRL-TK, miR NC+PDPK1 WT2+pRL-TK, miRNC+PDPK1 WT3+pRL-TK, and miR NC+UTR NC+pRL-TK, respectively. Forty-eight hours after transfection, the luminescent values of Firefly and Renilla luciferases activities were quantified using a Dual-Luciferase activity Assay Kit (Genomeditech, Shanghai, China) according to the manufacture's protocol, and each assay was performed in triplicate. Double luciferase reporter genes were tested to detect the transcription level of target gene to determine whether NF-κB was activated. The DF-1 cells were transfected with various plasmids and a pNF-κB-Luc plasmid (Genomeditech, Shanghai, China) together with a pRL-TK plasmid (Genomeditech, Shanghai, China) as an internal control. The test groups were si-NC+NF-κB-luc+TK, si-NC+NC-luc+TK, si-NC+PC-luc+TK, si-*PDPK1*+NF-κB-luc+TK, si-*PDPK1*+NC-luc+TK, and si-*PDPK1*+PC-luc+TK, respectively. Twenty-four hours after transfection, the cells were infected with ALV-J for 36 h, and then the cell extracts were assayed for luciferase activity using a dual luciferase reporter assay system (Genomeditech, Shanghai, China), according to the manufacturer's instructions. All experiments were performed in triplicate to verify the results.

### Electrophoretic Mobility Shift Assay

First, nuclear proteins were extracted from ALV-J infected cells, blank control cells, si-*PDPK1*+ALV-J cells, si-*PDPK1* (not vaccinated with ALV-J virus) cells, si-*PDPK1* NC+ALV-J cells, and si-*PDPK1* NC (not vaccinated with ALV-J virus) cells using nuclear protein extraction kit (Biotech, Shanghai, China). Next, the BCA protein quantification kit (Bebo, Shanghai, China) was used for protein quantification. Biotin-labeled electrophoretic mobility shift assay (EMSA) probe-NF-κB (0.2 μM) and EMSA probe-NF-κB (1.75 μM) were purchased from Beyotime Biotechnology, Shanghai, China. Binding reactions and separation on 6% non-denaturing polyacrylamide gels were performed according to the instructions in the EMSA kit (PIERCE, USA). Finally, protein electrophoresis, transfer, UV cross-linking, and chemiluminescence were used to detect biotin-labeled DNA.

### Cell Counting Kit 8 Assay

CEF were seeded into 96-well-plates at a density of 4 × 10^3^ cells/well and incubated for 24 h. The cells were then transiently transfected with gga-miR-148a-5p mimics, gga-miR-148a-5p mimics NC, gga-miR-148a-5p inhibitor, gga-miR-148a-5p inhibitor NC, PDPK1-siRNA, and control siRNA for various periods of time (12, 24, and 36 h). Then, 10 μl of ALV-J at a concentration of 10^6^ TCID50 was added to miR-148a-5p mimics [denoted as gga-miR-148a-5p-M (ALV-J+)], gga-miR-148a-5p mimics NC [denoted as gga-miR-148a-5p-NC (ALV-J+)], gga-miR-148a-5p-Inh-NC (ALV-J+) and miR-free (ALV-J+), and siRNA-*PDPK1* NC(ALV-J+) and siRNA-free (ALV-J+) for 36 h, respectively. Finally, cell counting kit-8 (CCK-8) (Meilun Biotechnology, Dalian, China) was performed to test the cell growth at 12, 24, and 36 transfection and 36 h of ALV-J postinfection according to manufacturer's protocol. In brief, at each time point, 10 μl CCK-8 reagent was added into each well. After 2 h of incubation at 37°C, the absorbance rates were measured at 450 nm by using a Microplate Reader (Thermo Fisher). All experiments were repeated at least three times.

### EdU Assay

In order to confirm the results of cell proliferation, the EDU method was adapted to test effects again. Similar to the CCK-8 method, the CEF cells were treated with gga-miR-148a-5p inhibitor, gga-miR-148a-5p mimics, or PDPK1-siRNA for different times. Each well was incubated with 50 μM EDU solution (RiboBio, Guhangzhou, China) at room temperature for 3 h and rinsed with PBS. And then, the cell plate was fixed with 4% paraformaldehyde (PFA), and cells were fixed with glycine solution and 0.5% Triton X-100 for about 10 min. Subsequently, the culture plate was immunostaining with Apollo staining reaction buffer, and the nuclei of cells were stained with DAPI. The images and quantities of EDU-stained cells (proliferating cells) were analyzed under fluorescence microscopy (Nikon, Tokyo, Japan). All the tests were triplicated.

### Flow-Cytometric Analysis of Cell Cycle

CEF cells were inoculated into six-well-plates and transfected with gga-miR-148a-5p mimics, gga-miR-148a-5p inhibitor, si-*PDPK1*, or NC. After incubating for 24 h, the CEF were vaccinated with ALV-J for 36 h, and cell cycle assays were performed. In brief, the medium was aspirated, and the cells were washed twice with PBS, digested, and collected and fixed with ice-cold 70% absolute ethanol for more than 18 h. Finally, cell staining was performed using a Cell Cycle Assay kit (Univ, Shanghai) and detection was performed using a BD AccuriC6 Flow cytometer (BD Biosciences). Cell cycle distribution was analyzed with the Flowjo10.6.1 software (https://www.flowjo.com/solutions/flowjo/downloads) and GraphPad Prism 5 software (San Diego, California, America). Each assay was performed in triplicate.

### Statistical Analysis

All experiment data were analyzed using GraphPad Prism 5.0 (San Diego, CA, USA), and all results are expressed as mean ± SD. All data were analyzed using SPSS 22.0 software (IBM, Chicago, IL, USA) for significant difference analysis. Significance was determined using least significant difference (LSD) test, and differences were considered significant at the *P* < 0.05 level.

## Results

### Screening for Differentially Expressed miRNA

**Figure 1:**
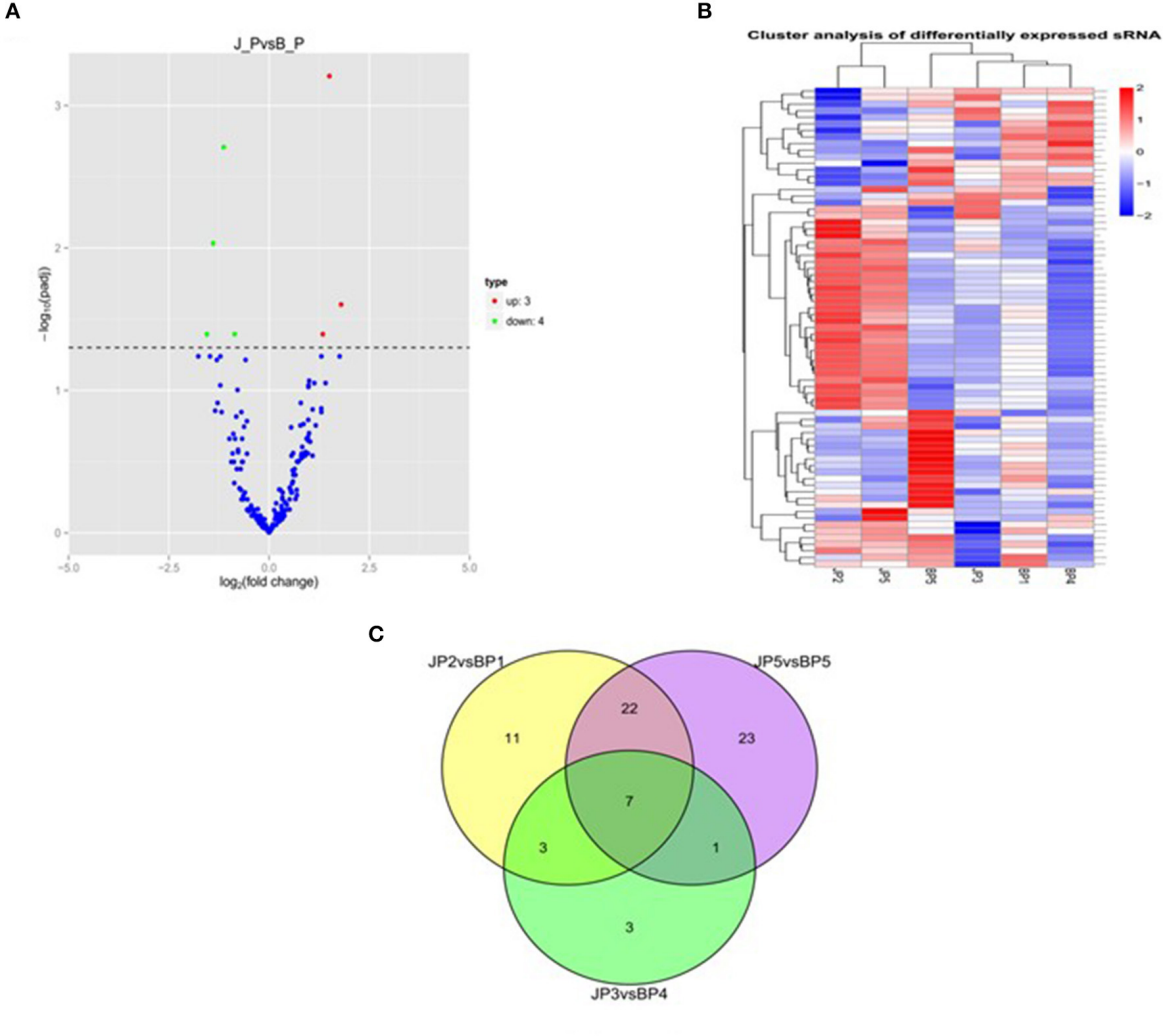
miRNA expression and differential analysis. **(A)** The difference in expression levels of miRNA between infected and uninfected chicken. The abscissa represents the fold change of miRNA expression in different experimental groups/different samples, and the ordinate represented the statistically significant degree of change in miRNA expression; scattered dots represent individual miRNAs: blue dots represent miRNAs with no significant difference, red dots indicate significantly upregulated differential miRNAs, and green dots indicate significantly downregulated differential miRNAs. **(B)** Heat map of differentially expressed miRNAs in each paired group (infected vs. uninfected). Red indicates high expression miRNA, blue indicates low expression miRNA. **(C)** Venn diagram of differential miRNA in six comparison groups. The large circles represent each comparison combination, the sum of the numbers in each large circle represent the total number of differential miRNAs of the comparison combination, and the overlapping parts of the circles represent the number of differential miRNAs in common between the combinations. BP represents the spleen tissue of blank control group; JP represents the spleen tissue of ALV-J-infected chicken.

To identify the major miRNAs involved in tumor disease in chicken, we screened differentially expressed miRNA between the infected and uninfected groups, based on our previous sequencing results of the spleen tissues of ALV-J-infected chickens and uninfected chickens at 40 days of age (Lan et al., 2017). Differentially expressed miRNAs (DEMs) were identified using fold change (FC) ≥2 and *P* < 0.05. A total of seven DEMs were detected. Of these, three upregulated and four downregulated miRNAs were detected in infected group relative to uninfected group (Figure 1A). Furthermore, clustering analysis indicated that the miRNA expression patterns were different in infected group and uninfected group (Figure 1B). In addition, there were 33 DEMs in different groups, of which seven were common among the three groups (Figure 1C); these seven differentially expressed miRNAs are shown in Table 3.

**Table 3:** Expression of seven differentially expressed miRNAs in ALV-J-infected chickens and uninfected chickens.

**sRNA**	**log^**2**^ fold change**	**pval**	**padj**	**Up/down**
gga-miR-148a-5p	−0.85949	0.0012164	0.040315	Down
gga-miR-203	−1.5466	0.0011138	0.040315	Down
gga-miR-205a	1.798	0.00043007	0.024944	Up
gga-miR-21-3p	−1.1313	1.69E−05	0.0019577	Down
gga-miR-21-5p	−1.3936	0.00011952	0.009243	Down
gga-miR-223	1.34	0.00096377	0.040315	Up
gga-miR-383-5p	1.5067	2.68E−06	0.00062092	Up

### Analysis of gga-miR-148a-5p Structure and Candidate Gene Identification

**Figure 2:**
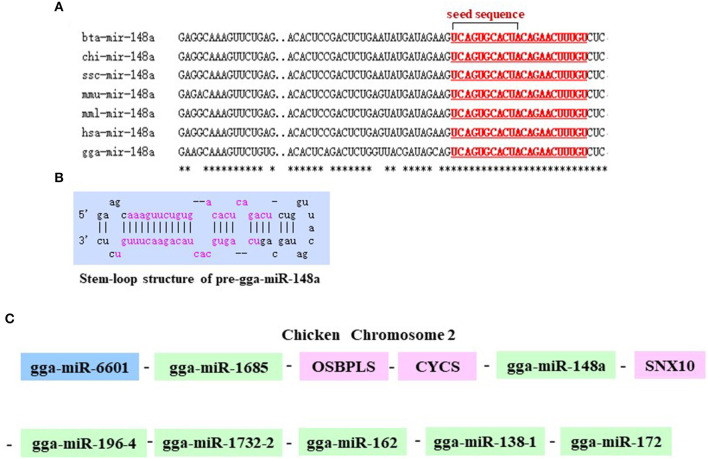
Chromosomal position of gga-miR-148a and comparison of the gga-miR-148a sequences among several species. **(A)** Seed sequences of different species gga-miR-148a-5p. **(B)** Stem-loop structure of pre-gga-miR-148a-5p. **(C)** gga-miR-148a-5p is located on chicken chromosome 2.

**Figure 3:**
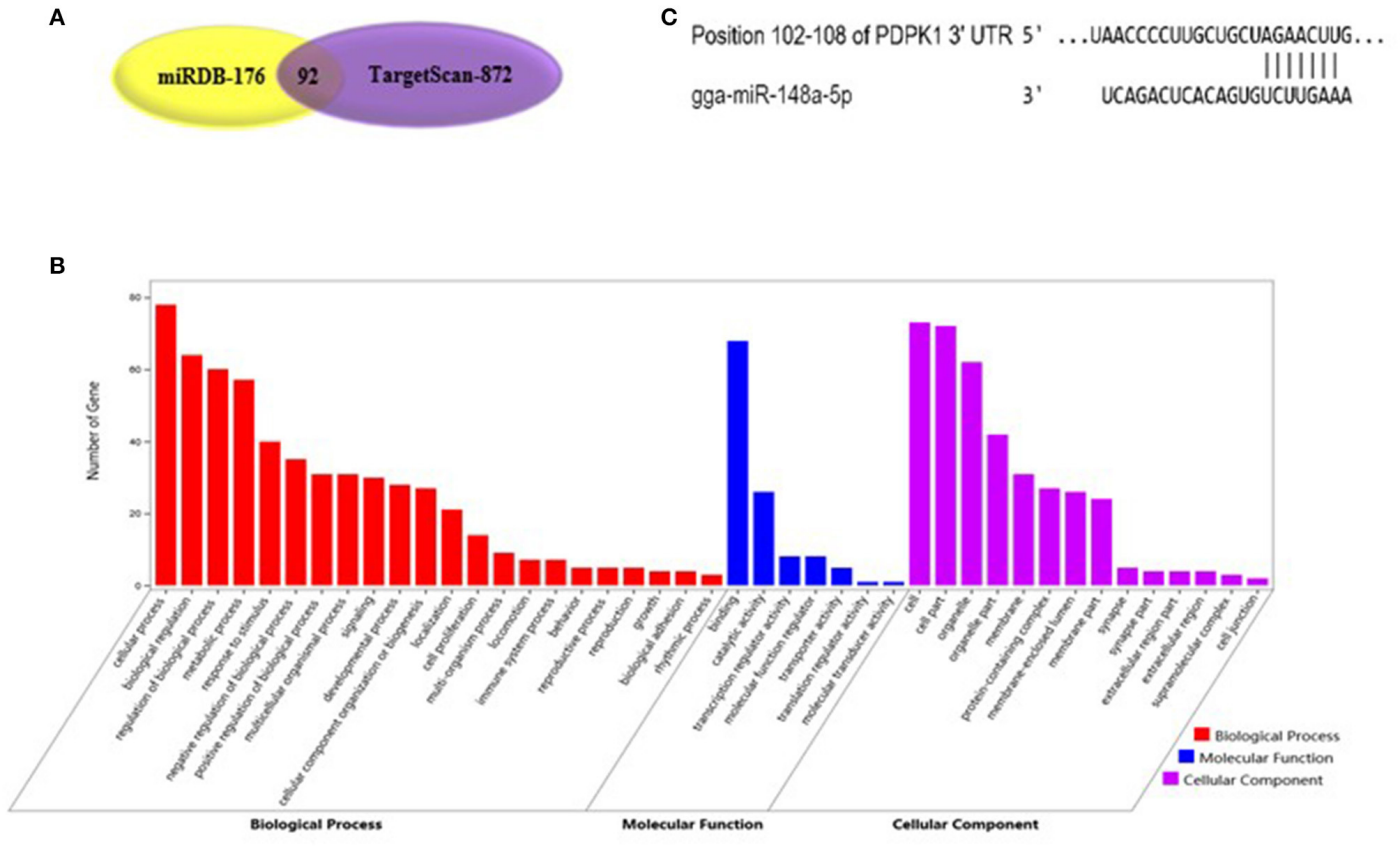
Functional enrichment analysis of target genes. **(A)** The prediction of target genes using TargetScan and miRDB. **(B)** Histogram of GO enrichment of gga-miR-148a-5p intersection target genes. The horizontal axis represents the GO term of enrichment of differential genes, and the vertical axis represents the number of differential genes; red indicates biological process, blue indicates molecular function, and purple indicates cell component. **(C)** gga-miR-148a-5p and its potential target gene PDPK1 3′-UTR binding site analysis.

As shown in Figure 2A, gga-miR-148a-5p seed sequence was highly conserved among seven species including cow (*Bos Tauru*s), pigeon (*Columba livia*), pig (*Sus Scrof* a), mouse (*Mus musculus*), monkey (*Macaca mulatta*), human (*Homo sapiens*), and chicken (*Gallus gallus*). In chicken, stem-loop structure of pre-gga-miR-148a is shown in Figure 2B. The gga-miR-148a-5p was located between *CYCS* and *SNX10*, on chromosome 2, as shown in Figure 2C. To better understand potential gga-miR-148a-5p functions, a total of 341 and 872 target genes of gga-miR-148a-5p were identified through miRDB and Targetscan database based on the gga-miR-148a-5p sequence, respectively, of which, 92 target genes were shared by the two databases. First, the 92 intersected target genes were enriched in the NF-κB signaling pathway through David (https://david.ncifcrf.gov/) biological information network, and *PDPK1* was enriched in the NF-κB signaling pathway. To filter for tumor-associated targets, we used the OmicShare platform (www.omicshare.com) to perform GO enrichment analysis on 92 intersected target genes of gga-miR-148a-5p (Figure 3A). As shown in Figure 3B, Table 4, the intersection target genes of gga-miR-148a-5p were enriched to 2,789 GO terms, and 111 GO terms were significantly enriched (*P* < 0.01) in tumorigenesis mechanism-related terms, including regulation of cellular process (GO:0050794), regulation of biological processes (GO:0050789), and viral RNA genome replication (GO:0039694). Of the 111 GO terms, four genes were involved in immune-related pathways (GO:0043122): 3-phosphoinositide-dependent protein kinase 1 (*PDPK1*), mitogen-activated protein kinase kinase kinase 2 (*MAP3K2*), ankyrin repeat domain 17 (*ANKRD17*), and TGF-beta-activated kinase 1/MAP3K7-binding protein 3 (*TAB3)*. All four genes were highly enriched in NF-κB signaling pathway. Therefore, *PDPK*1 was selected for further experimental validation. At the same time, through GeneCards (https://www.genecards.org/) analysis, we identified that PDPK1 (PDK1) was involved in three signaling pathways including AKT signaling pathway, NF-κB pathway, and T cell receptor signaling pathway. Because NF-κB plays a key role in regulating the immune response to infection, therefore, we choose this pathway as the research object. Subsequently, through PathCards (https://pathcards.genecards.org/) analysis, we found that there were 101 genes in the NF-κB pathway (Supplemental Table S1) and PDPK1 was one of them. Finally, through the Targetscan (www.targetscan.org/vert_72/) and miRbase (www.mirbase.org/), it was found that gga-miR-148a-5p and *PDPK1* 3′-UTR had complementary binding sites (Figure 3C), and the context score ≤ 0.2.

**Table 4:** GO enrichment analysis of gga-miR-148a-5p target genes.

**GO ID**	**GO term**	**Gene numbers**	***P*-value**	**Gene name**
GO:0043412	Macromolecule modification	33	0.0002	UBE2G1, PDPK1, ATG16L1, etc.
GO:0048519	Negative regulation of biological process	35	0.0005	TMOD3, PDPK1, MAP3K2, etc.
GO:0044260	Cellular macromolecule metabolic process	51	0.0006	RNF111, PDPK1, MAP3K2, etc.
GO:0019538	Protein metabolic process	40	0.0006	RNF111, PDPK1, MAP3K2, etc.
GO:0043170	Macromolecule metabolic process	56	0.0008	CPEB3, PDPK1, HSPA5, etc.
GO:0060255	Regulation of macromolecule metabolic process	38	0.0015	RNF111, PDPK1, MAP3K2, etc.
GO:0065007	Biological regulation	64	0.0019	OSBPL8, PDPK1, RBPJ, etc.
GO:0019222	Regulation of metabolic process	39	0.0032	TP53INP2, PDPK1, USP7, etc.
GO:0050789	Regulation of biological process	60	0.0035	CACNA1D, PDPK1, MAP3K2, etc.
GO:0048523	Negative regulation of cellular process	30	0.0037	TMOD3, PDPK1, MAP3K2, etc.
GO:0051171	Regulation of nitrogen compound metabolic process	35	0.0052	TP53INP2, PDPK1, CTCF, etc.
GO:0080090	Regulation of primary metabolic process	35	0.0074	RNF111, PDPK1, MAP3K2, etc.
GO:0005634	Nucleus	44	0.0002	HSPA5, TP53INP2, CTCF, etc.
GO:0043227	Membrane-bounded organelle	58	0.0016	SLC25A36, PDPK1, MAP3K2, etc.
GO:0043231	Intracellular membrane-bounded organelle	55	0.0022	HSPA5, GOLGA1, TP53INP2
GO:0005737	Cytoplasm	55	0.0025	CPEB3, PDPK1,MAP3K2, etc.
GO:0043229	Intracellular organelle	62	0.0039	CACNA1D, PDPK1,MAP3K2, etc.
GO:0044424	Intracellular part	68	0.0078	HSPA5, GOLGA1, TP53INP2, etc.
GO:0043226	Organelle	62	0.0098	CACNA1D, PDPK1, MAP3K2, etc.

*The table only lists the results of the analysis with the highest number of genes, and the P-value has been corrected by FDR (false-discovery rate)*.

### Expression of gga-miR-148a-5p and *PDPK1* Gene in the ALV-J-Infected CEF

**Figure 4:**
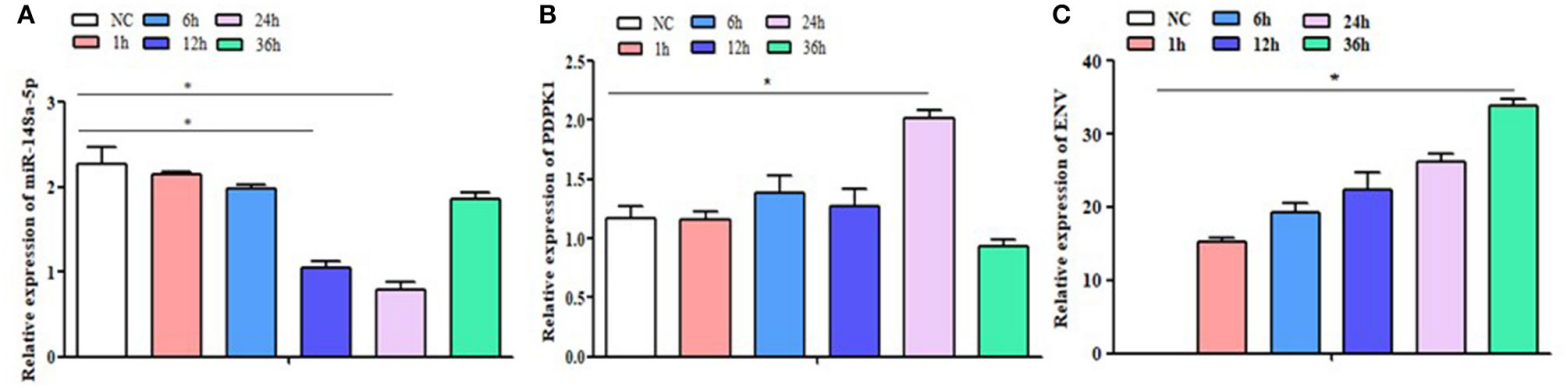
NC: the control group without ALV-J virus infection; 1 h, 6 h, 12 h, 24 h and 36 h: the different time after infection with ALV-J virus. The expression level of gga-miR-148a-5p, *PDPK1*, and ENV gene in the CEF cells at different infection times. Significant differences in gene expression levels between control and treatment (ALV-J infection at different time) are indicated as follows: *P < 0.05. Error bars indicate the SEM of replicates performed in triplicate.

To determine the expression of gga-miR-148a-5p and its putative target gene, *PDPK1* gene associated with ALV-J induction, qRT-PCR was performed using cDNA of CEF cells from ALV-J-infected and control groups. As shown in Figure 4A, the gga-miR-148a-5p expression was downregulated after ALV-J infection. Especially at 12 and 24 h postinfection, the gga-miR-148a-5p levels were significantly decreased when compared with noninfected CEF cells (*P* < 0.05). Conversely, the *PDPK1* expression in CEF cell was upregulated after ALV-J infection except for 36 h after infection. Meanwhile, *PDPK1* gene expression level was significantly higher than that of the control group at 24 h after infection (*P* < 0.05), but no significant difference was observed at other time points (Figure 4B). In addition, the expression level of virus gene *ENV* was upregulated with the increase of time, especially at 36 h, the *ENV* expression level was significantly higher than that of the control group (*P* < 0.05) (Figure 4C). These results indicated that *PDPK1* mRNA expression level was negatively correlated with gga-miR-148a-5p in ALV-J-infected CEF cells.

### The Target Regulation Relationship Between gga-miR-148a-5p and *PDPK1* Gene

**Figure 5:**
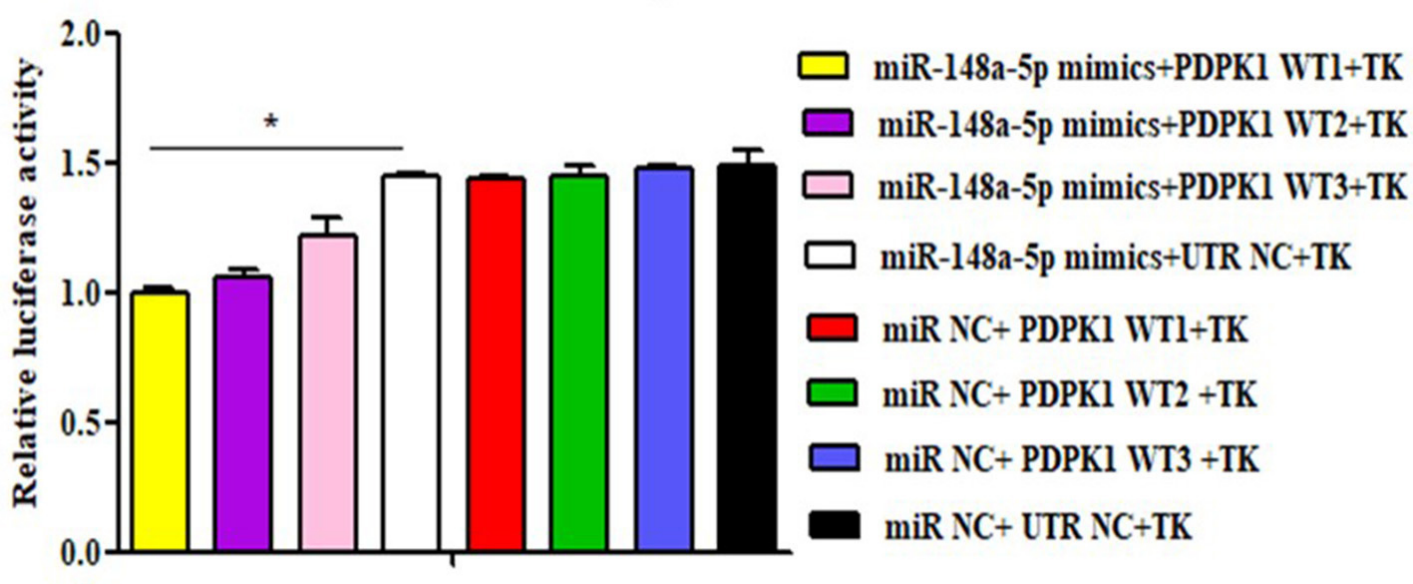
Luciferase reporter assays in HEK 293 cells transfected with reporter vectors containing either the wild-type or blank plasmid vector. TK indicates renal internal reference; UTR NC indicates control for constructing 3′-UTR reporter gene plasmid. The data are expressed as the mean ± SEM. *P < 0.05.

To further verify whether gga-miR-148a-5p was able to combine with *PDPK1* 3′-UTR sequence, a luciferase reporter assay was performed using the HEK 293 cell line. Three wild-type *PDPK1*-3′-UTR-containing putative binding sites were separately cloned into the PGL3-CMV-LUC-MCS dual luciferase reporter vector. The first one (*PDPK1*-WT1) was about 200 bp upstream and downstream of the 42 sites, the second one (*PDPK1*-WT2) was about 200 bp upstream and downstream of the 2,455 sites, and the third one (*PDPK1*-WT3) was about 200 bp upstream and downstream from the 3,731 sites to the 4,110 sites. Then, we cotransfected HEK 293 cells with the gga-miR-148a-5p mimic together with the reporter vector containing the wild type. As shown in Figure 5, compared with the control group, the luciferase activity was reduced when the gga-miR-148a-5p was cotransfected with the three wild-type *PDPK1* 3′-UTR-containing vector. Especially for *PDPK1*-3′U W1, the luciferases activity was significantly reduced (*P* < 0.05). In contrast, cotransfection of blank vector containing wild-type did not affect the luciferase activity (*P* > 0.05). These results demonstrated that miR-148a-5p can target and bind to the 3′-UTR of *PDPK1* gene and regulate its expression.

### gga-miR-148a-5p Negatively Regulates PDPK1 Expression

**Figure 6:**
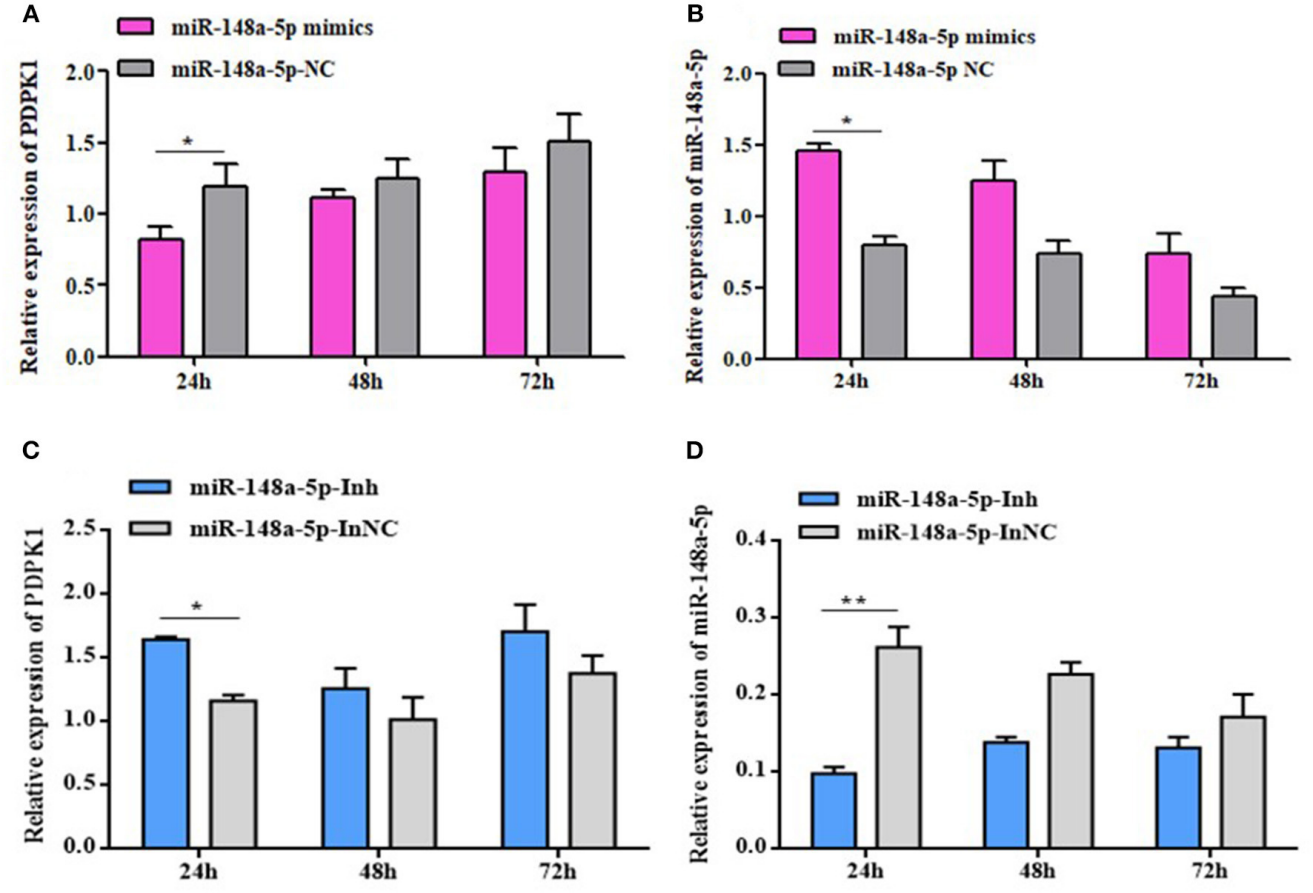
gga-miR-148a-5p regulates *PDPK1* expression. **(A)** The expression level of *PDPK1* in CEF cells overexpression gga-miR-148a-5p is reduced. **(B)** The expression level of gga-miR-148a-5p in CEF cells overexpression gga-miR-148a-5p is increased. **(C)** Transfection with gga-miR-148a-5p inhibitor increased the mRNA expression of *PDPK1* in CEF cells. **(D)** Transfection with gga-miR-148a-5p inhibitor decreased the mRNA expression of gga-miR-148a-5p in CEF cells. All of the error bars represent the means ± SEM of triplicated experiments. One-way ANOVA was used to analyze the significant differences. *P < 0.05; **P < 0.01.

To fully validate the impact of interaction between gga-miR-148a-5p and *PDPK1*, we tested the effects of overexpression or inhibition of gga-miR-148a-5p on *PDPK1* expression in CEF cells. The results showed that the expression level of *PDPK1* was reduced at 24, 48, and 72 h postmimic transfection. Especially at 24 h, the *PDPK1* expression level was significantly induced compared with the control group (*P* < 0.05) (Figure 6A). On the contrary, the expression level of gga-miR-148a-5p was significantly increased in the overexpression group compared with the negative control (Figure 6B). Furthermore, the gga-miR-148a-5p inhibitor inhibited the expression of gga-miR-148a-5p noticeably (Figure 6D), leading to an increase in *PDPK1* mRNA levels (Figure 6C). Especially at 24 h postinhibitor transfection, the gga-miR-148a-5p and *PDPK1* expression levels were significantly decreased and increased, respectively, compared with the negative control (*P* < 0.01 and *P* < 0.05). These results suggested that there was a negative correlation between the expression of gga-miR-148a-5p and the *PDPK1* gene.

### gga-miR-148a-5p Inhibits Cell Proliferation and Cycle Progression of ALV-J-Infected CEF Cells

**Figure 7:**
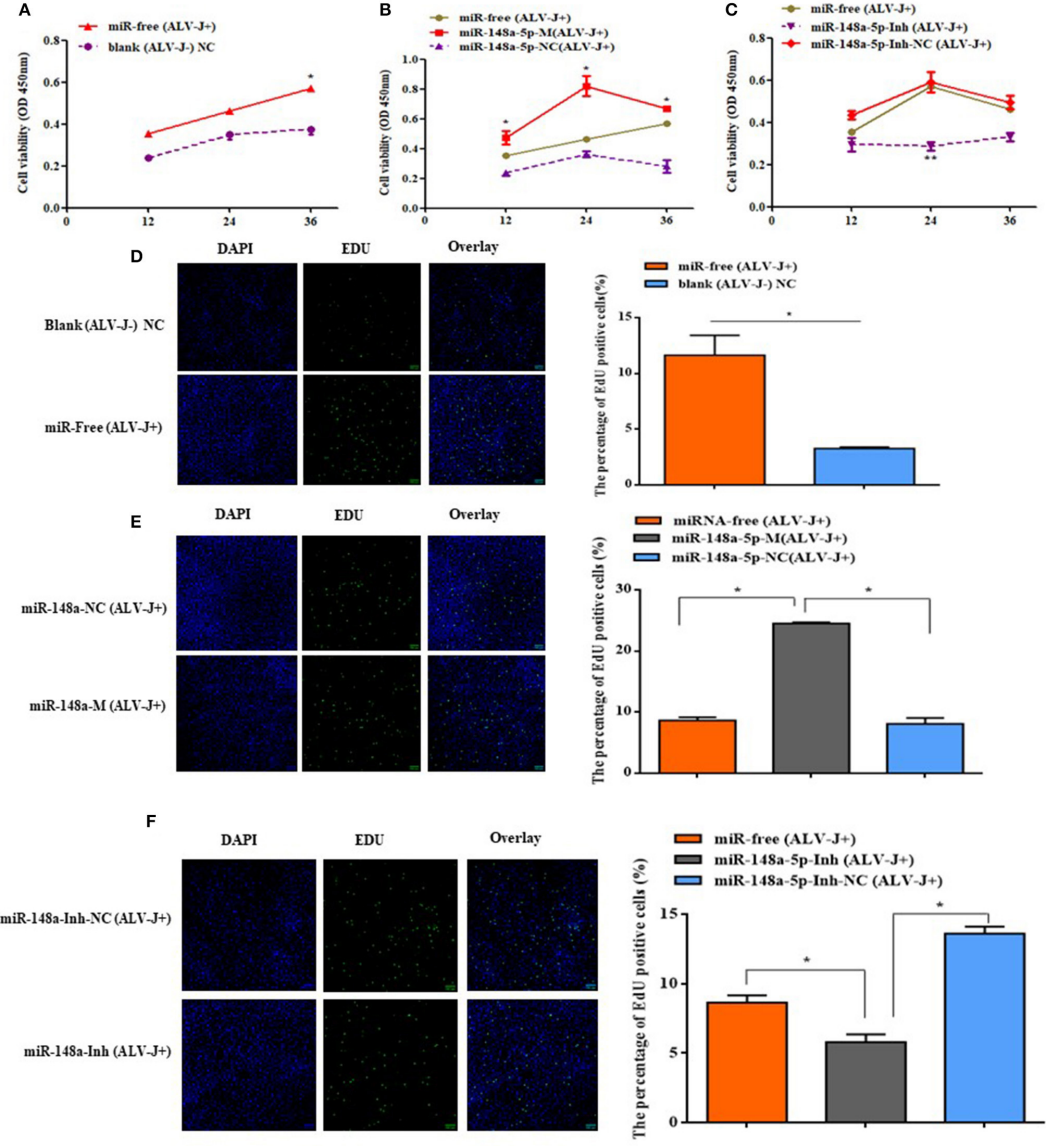
Cell proliferaction was influenced by gga-miR-148a-5p. **(A)** Cell multiplication was promoted with ALV-J affection. **(B,C)** Effect of gga-miR-148a-5p on ALV-J-infected CEF cell proliferation. Cell proliferation was detected by a CCK-8 assay at 12, 24, and 36 h after transfection, and then 36 h of ALV-J postinfection, with the gga-miR-148a-5p mimics, inhibitor, and respective NC (*n* = 3). **(D)** EdU assay for CEF infected with ALV-J; **(E,F)** EdU assay for CEF transfected with gga-miR-148a-5p mimic, inhibitor, and respective NC. EdU (green) fluorescence is used as an indicator of proliferation, and nuclei are indicated by DAPI (blue) fluorescence; micrographs were taken using ×200 magnification. All the error bars represent the means ± SEM of triplicated experiments. *P < 0.05.

The effects of gga-miR-148a-5p on ALV-J-infected CEF cell proliferation and cycle were investigated by CCK-8, EdU assays, qRT-PCR, and flow cytometry. CCK-8 assay results showed that the cell multiplication of the miR-free (ALV-J+) group was significantly promoted at 36 h compared with the blank (ALV-J–) group (*P* < 0.05; Figure 7A). Meanwhile, we also found that overexpression of gga-miR-148a-5p-M (ALV-J+) significantly increased the proliferation of CEF cells at 12, 24, and 36 h posttransfection and then 36 h of ALV-J postinfection compared with the gga-miR-148a-5p-NC (ALV-J+) and miR-free (ALV-J+) groups (Figure 7B). Furthermore, the effects of the gga-miR-148a-5p inhibitor on cell proliferation were assayed using the same grouping. Compared with the gga-miR-148a-5p-Inh-NC (ALV-J+) and miR-free (ALV-J+) groups, the proliferation of CEF cells of gga-miR-148a-5p-Inh (ALV-J+) was significantly decreased at 24 h posttransfection (Figure 7C). In order to verify the above results, we also used the EdU assay and found that the number of EdU-positive cells in the miR-free (ALV-J+) group was significantly higher than that of blank (ALV-J–) group (*P* < 0.05; Figure 7D), and the EdU-positive cell number in the proliferation period was significantly increased by gga-miR-148a-5p overexpression and decreased by gga-miR-148a-5p inhibitor (Figures 7E,F). Therefore, these results suggested that gga-miR-148a-5p inhibits CEF cell proliferation in ALV-J infection.

**Figure 8:**
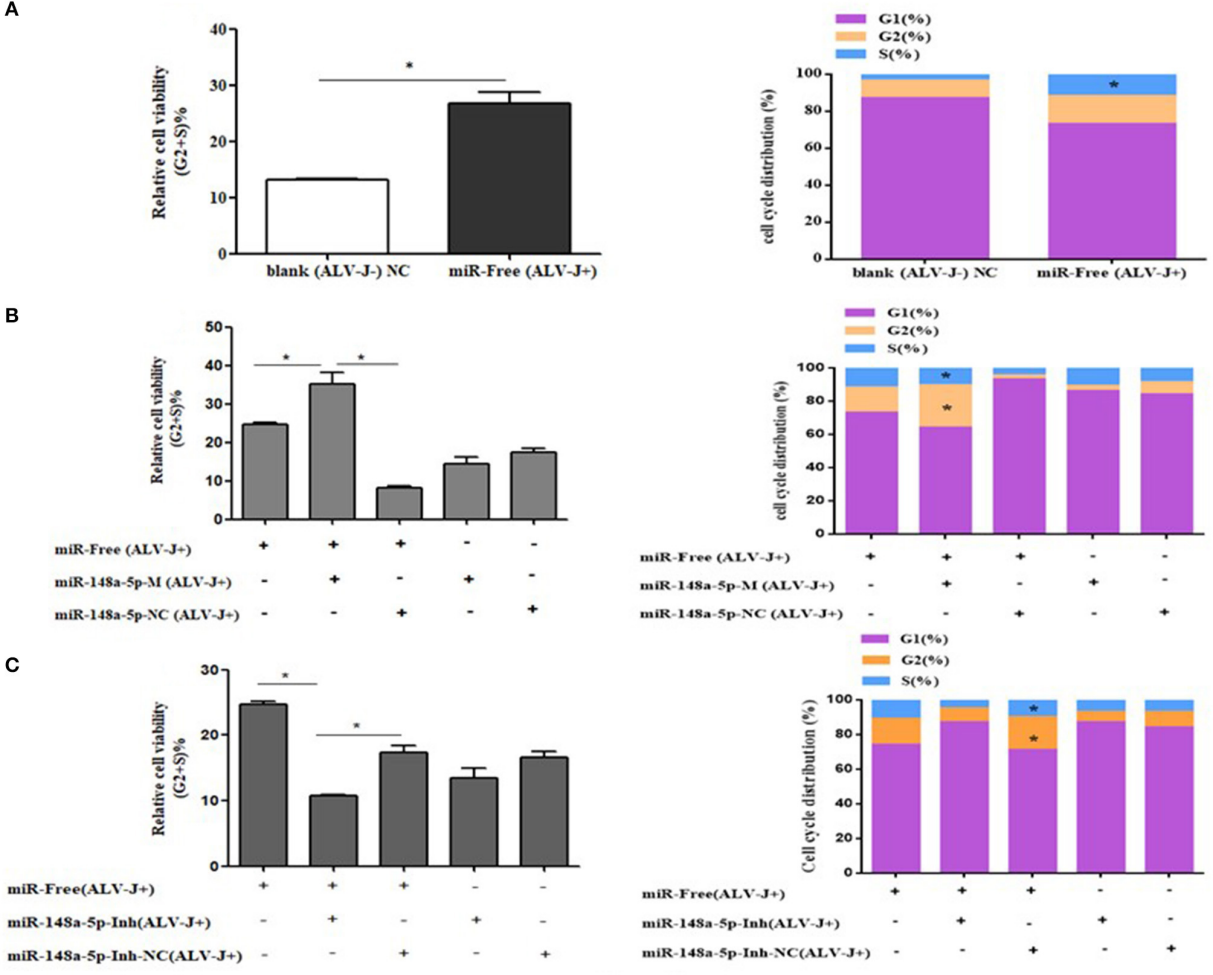
Cycle procession of ALV-J-infected CEF cells was affected by gga-miR-148a-5p. **(A)** Cell cycle was promoted with ALV-J-infected cells. **(B)** Cell cycle was dramatically promoted with gga-miR-148a-5p overexpression. **(C)** Cell cycle was suppressed by gga-miR-148a-5p inhibitor. Values are mean means ± SEM. *P < 0.05.

To further explore how gga-miR-148a-5p inhibited CEF cell proliferation, flow cytometry was performed to detect the effect of gga-miR-148a-5p on cell cycle distribution. As mentioned above, CEF cells were transfected with synthetic RNA oligonucleotides and cocultured with gga-miR-148a-5p mimics or gga-miR-148a-5p inhibitor for 24 h. Then 200 μl of ALV-J (10^6^ TCID50) were added to each well for 36 h. We demonstrated that compared with the blank (ALV-J–) group, the proportion of cells in phase G2 and S was significantly increased in the miR-free (ALV-J+) group, and the proportion of G1 phase decreased (Figure 8A). At the same time, overexpression of gga-miR-148a-5p increased the proportion of cells in the G2+S phase but decreased the proportion of cells in the G1 phase compared with the gga-miR-148a-5p NC (ALV-J+) and miR-free (ALV-J+) groups (Figure 8B). In contrast, the proportion of G1 phase cells increased significantly after gga-miR-148a-5p inhibitor compared with the miR-148a-Inh NC (ALV-J+) and miR-free (ALV-J+) groups (Figure 8C). Taken together, these results suggested that gga-miR-148a-5p inhibits the proliferation of CEF cells by inhibiting cell cycle progression.

### PDPK1 Knockdown Promotes CEF Cell Proliferation and Cycle Progression

**Figure 9:**
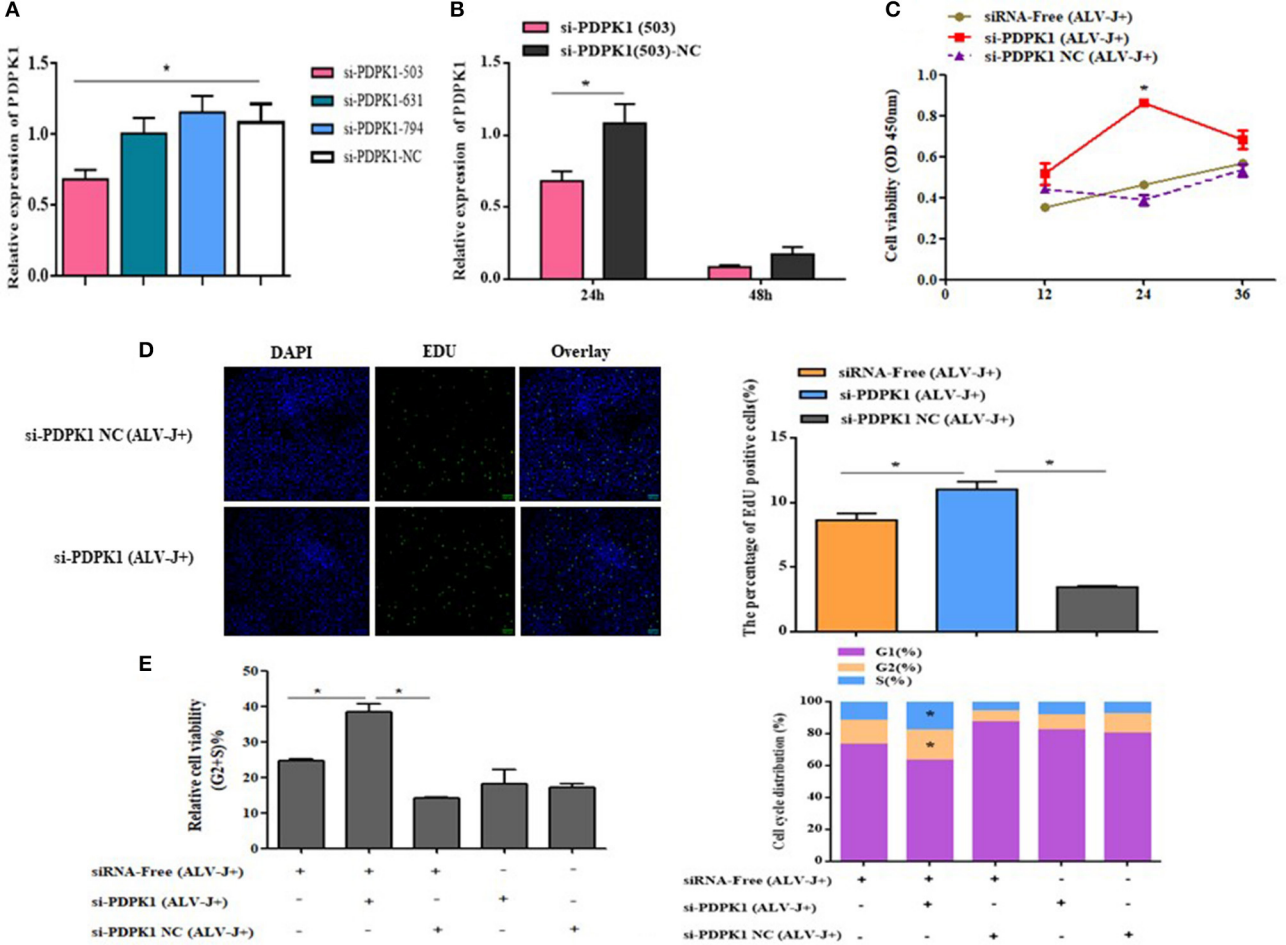
The effect of knockdown of *PDPK1* on the proliferation and cell cycle progression of ALV-J-infected CEF cell. **(A,B)** Screening *PDPK1* interfering fragments and verifies the *PDPK1* relative expression after transfected si-*PDPK1* (503). **(C)** Cell growth curves measured by CCK-8 assays following transfection with si-*PDPK1* and negative control in ALV-J-infected CEF cells. **(D)** The results of the EdU assay for CEF cells transfected with si-*PDPK1* and negative control, where EdU (green) fluorescence is used as an indicator of proliferation, and nuclei are indicated by DAPI (blue) fluorescence, micrographs were taken using ×200 magnification. **(E)** Cell cycle was promoted with *PDPK1* knockdown. All of the error bars represent the means ± SEM of triplicated experiments. *P < 0.05.

In order to determine the role of *PDPK1* in the proliferation and cycle of ALV-J-infected CEF cells, we designed three siRNAs to interfere with *PDPK1* expression. As the interference efficiency of siRNA-*PDPK1*-503 was the highest at 24 h (Figure 9A), siRNA-*PDPK1*-503 (hereafter called siRNA-*PDPK1*) was selected for further study. Its interference efficiency was 62% at 24 h and 47% at 48 h posttransfection (Figure 9B). The cellular proliferation viability was significantly increased in siRNA-*PDPK1* (ALV-J+) group at 24 and 36 h post-siRNA transfection compared with siRNA-*PDPK1* NC(ALV-J+) and siRNA-free (ALV-J+) group (Figure 9C). Consistent with CCK-8, the EdU results showed that the number of EdU-positive cells in the siRNA-*PDPK1* (ALV-J+) group was significantly higher than that of siRNA-*PDPK1* NC(ALV-J+) and siRNA-free (ALV-J+) groups (*P* < 0.05) (Figure 9D). Furthermore, the flow cytometry results showed that the proportion of cells in G2+S phase was increased and the proportion of cells in G1 phase was decreased (Figure 9E). These findings revealed that knockdown *PDPK1* can promote ALV-J-infected cell proliferation and cycle progression.

### PDPK1 Had No Effect on NF-κB Activity

**Figure 10:**
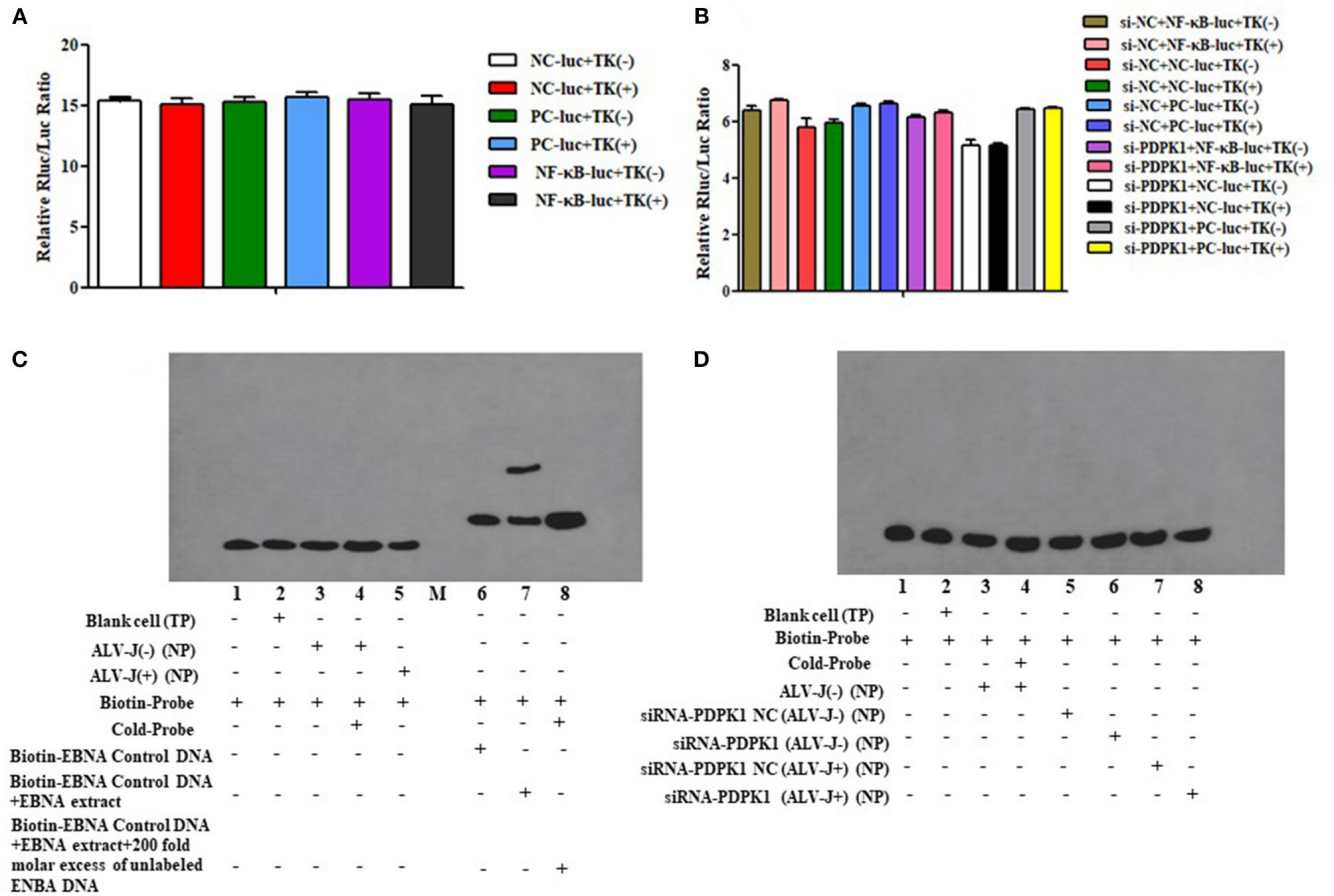
The effect of knockdown of *PDPK1* on NK-κB activity of ALV-J-infected CEF cell. **(A,B)** The effect of ALV-J and knockdown of *PDPK1* on NK-κB activity of CEF cell using dual luciferase reporter gene test, respectively. **(C,D)** The effect of ALV-J and knockdown of *PDPK1* on NK-κB activity of CEF cell using EMSA, respectively.

Previous studies have demonstrated that *PDPK1* activated the NK-κB signaling pathway in human gastric cancer (Wu et al., 2017). In order to explore the function of *PDPK1* in chicken leukemia disease, we detected NK-κB activity in ALV-J-infected CEF cells after si-*PDPK1* transfection using a NF-κB luc plasmid. As shown in Figures 10A,B, we found that inhibiting *PDPK1* in CEF cells had no effect on NK-κB activity. For further verification of this result, we performed using EMSA assays again. We found that NK-κB activity was not activated in CEF cell after ALV-J infection (Figure 10C). This result was consistent with the results of the dual luciferase reporter assay. In ALV-J-infected CEF cells after si-*PDPK1* transfection, the EMSA results showed that si-*PDPK1* had no significant effect on NK-κB activity in ALV-J-infected CEF compared with the control group (Figure 10D).

## Discussion

ALV-J avian leukemia is one of the most infectious pathogens among avian viral disease, which has caused significant economic losses to the poultry farming industry and seriously threatens the prosperity of the poultry industry worldwide (Cui et al., 2009; Payne and Nair, 2012). miRNAs are short non-coding RNAs that have been shown to play an important role in regulation of various biological process and the innate and adaptive immunity of the host in recent years (Bartel, 2004; Braconi et al., 2010; Fiorucci et al., 2015). Recently, with the application of high-throughput sequencing and gene chip technology, more and more miRNAs related to avian diseases have been discovered. For example, gga-miR-375 may participate in tumorigenesis induced by subgroup J Avian leucosis virus (Li H. et al., 2014). gga-miR-2127 plays pivotal roles in weakening the antiviral innate immune response by targeting bursa virus (Ouyang et al., 2017). Previously, our miRNA sequencing data indicated that gga-miR-148a-5p expression level was significantly different in ALV-J-infected chicken spleens compared with uninfected chicken (Lan et al., 2017). However, the role of gga-miR-148a-5p in ALV-J-infected chickens and the underlying mechanisms are still largely unclear.

It is well-known that the development of tumors is often associated with cell proliferation and apoptosis (Chu et al., 2017). Previous studies demonstrated that the miR-148a was considered an important tumor suppressor by inhibiting cell proliferation, apoptosis, and migration. Li et al. found that miR-148a targeted B cell lymphoma 2 (Bcl-2) to decrease the cell growth and increase apoptosis of breast cancer (Li Q. et al., 2017). Additionally, the research from Murata et al. exhibited that miR-148a could inhibit cancer cell proliferation in prostate cancer (Murata et al., 2010). Until now, the research on gga-miR-148a is very limited in chickens. Therefore, whether gga-miR-148a can reduce the incidence of tumor in chickens by inhibiting cell proliferation and cycle is still unknown. To our knowledge, this study is the first study to demonstrate that gga-miR-148a-5p may be involved in the pathogenesis of ALV-J, as it appears to influence many aspects of tumor formation, including cell proliferation and cell cycle progression. We found that ALV-J infection decreased the expression of gga-miR-148a-5p in CEF cells, especially at 12 and 24 h postinfection. Meanwhile, overexpression of gga-miR-148a-5p promoted cell proliferation, while knockdown of gga-miR-148a-5p inhibited cell proliferation, indicating that gga-miR-148a-5p has an important role during ALV-J infection. We found that gga-miR-148a-5p overexpression increased the proportion of cells in G2+S phase and reduced the proportion of cells in G1 phase, while the opposite results appeared after interference with gga-miR-148a-5p, suggesting that it can inhibit cell cycle progression.

From previous studies, it is not difficult to see that most of the researches on miRNA have focused on their ability to regulate cellular processes by targeting different genes, while many current studies also show that a single miRNA can have multiple target genes (Kim et al., 2008; Cao et al., 2020), as is the case with gga-miR-148a-5p. In order to explore which genes was targeted by gga-miR-148a-5p to mediate the proliferation and cell cycle of ALV-J-infected CEF cells, bioinformatics analysis was performed using two different software tools, TargetScan and miRDB, and 92 most likely target gene were identified. Among these genes, the *PDPK1* gene is of particular concern. *PDPK1* (namely *PDK1*) is an ancient serine-threonine kinase belonging to the AGC kinase family and has properties as a major regulator of AGC kinase, which mediates intracellular signaling such as cell growth, survival, and gene expression (Vanhaesebroeck and Alessi, 2000). Recently, research demonstrated that *PDPK1* is associated with cancer and tumors. For example, Zheng et al. found that deficiency of *PDPK1* significantly reduced the proliferation and migration of hemangioendothelioma endothelial cells (EOMA cells) (Zheng et al., 2015). Furthermore, in other tumor models, the proliferation and progression of cells can be reduced by knockdown of *PDPK1* (Ye et al., 2015). All of these findings showed that *PDPK1* may be a tumor suppressor. However, whether *PDPK1* has tumor suppressive or carcinogenic effects in tumors and cancers is still controversial (Bayascas et al., 2005). Different from previous studies in human cancer, our data show that *PDPK1* knockdown promotes CEF cell proliferation and cell cycle progression after ALV-J infection. Therefore, we speculate that this may be related to the characteristics of ALV-J virus and the specific immune system of birds. Subsequently, we confirmed that *PDPK1* was a target gene of gga-miR-148a-5p by the dual luciferase reporter assay and qRT-PCR results in the current study. Meanwhile, results of qRT-PCR showed that the low expression of gga-miR-148a-5p could promote the mRNA expression of *PDPK1*, indicating that there is a negative correlation between gga-miR-148a-5p and *PDPK1*. In addition, these findings suggest that gga-miR-148a-5p can regulate the antiviral capacity of *PDPK1* by promoting the translation of *PDPK1* mRNA. Therefore, gga-miR-148a-5p is a potential interfering target, which can promote the expression of *PDPK1* to inhibit cell proliferation and improve the host's immunity to virus infection and tumor formation.

**Figure 11:**
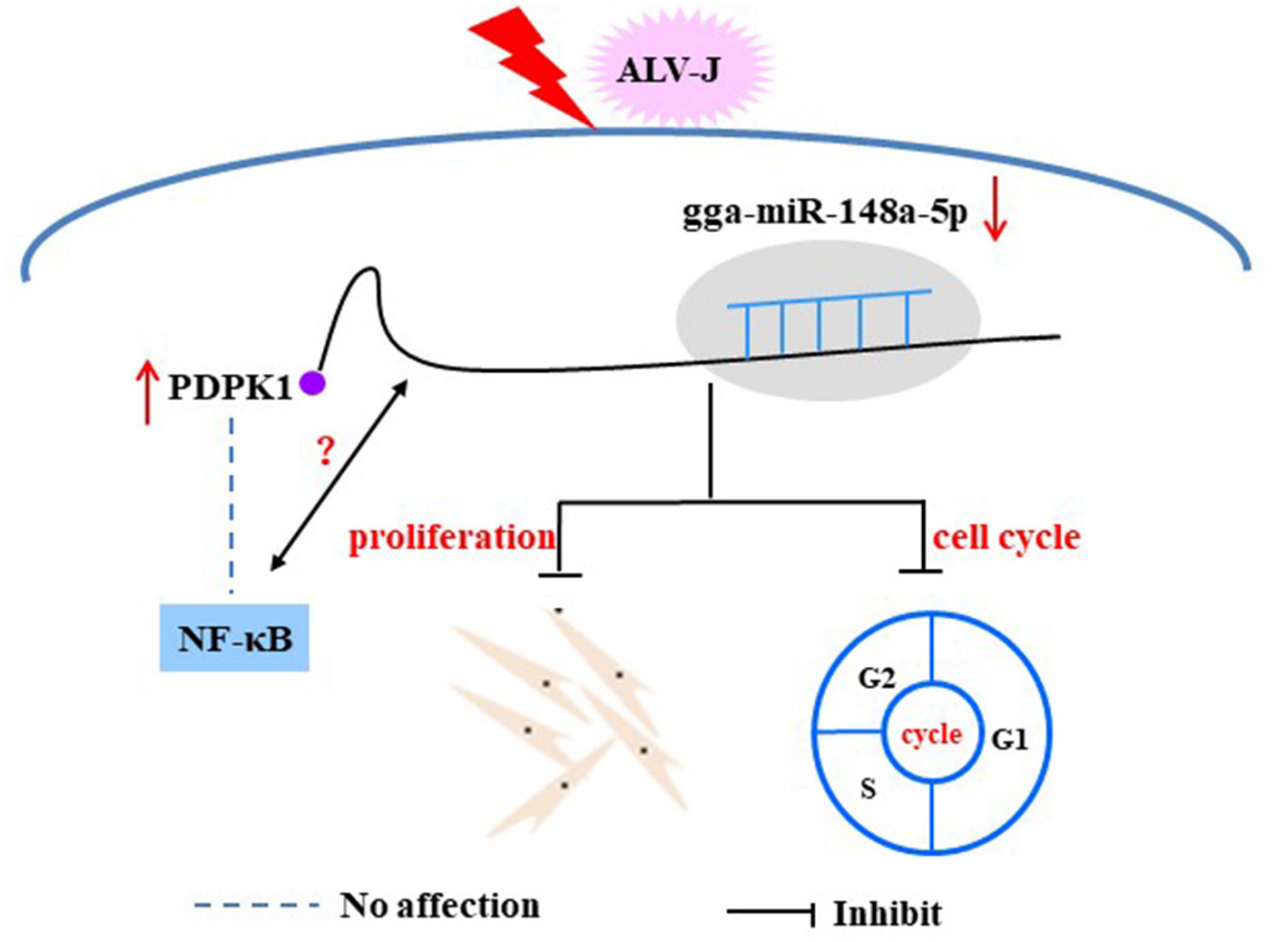
Proposed model of gga-miR-148a-5p regulation on ALV-J-infected CEF cell proliferation and cell cycles.

NF-κB, as an important transcription factor is mainly involved in regulating the response to inflammation, cell proliferation, and apoptosis (Tanaka et al., 2005; Hayden and Ghosh, 2011). A growing body of research suggests that many miRNAs can regulate viral replication through influencing NF-κB activity. For instance, Tian and He have found that has-miR-215 can block the NF-κB signaling pathway by targeting the tripartite motif 22 (TRIM22), thus playing a positive regulatory role on hepatitis C virus (HCV) replication (Tian and He, 2018), and Tanaka et al. found that overexpression *PDK1* could induce c-Myc and cyclin D expression via activating NF-κB. Thus, *PDK1* is considered a promising and attractive chemotherapy target for tumors (Tanaka et al., 2005). However, to our knowledge, there is no related study on the involvement of *PDPK1* in ALV-J-infected chicken by regulating NF-κB signaling. In this study, we showed that interference with *PDPK1* had no effect on the activity of NF-κB. These results demonstrate that *PDPK1* cannot directly affect NF-κB to regulate ALV-J infection in chickens. In conclusion, our study identified that ALV-J infection induces gga-miR-148a-5p expression. Meanwhile, gga-miR-148a-5p could inhibit ALV-J-infected CEF cell proliferation and cell cycle progression by negatively regulating *PDPK1* (Figure 11). These results may provide fundamental information for improving poultry disease resistance.

## Data Availability Statement

The raw data supporting the conclusions of this article will be made available by the authors, without undue reservation.

## Ethics Statement

The animal study was reviewed and approved by Institutional Animal Care and Use Committees of Sichuan Agricultural University.

## Author Contributions

HYu, SZ, CY, YLu, and QH performed experiments. HYu and YW wrote the original draft. XL and DL analyzed the data. HYi, XZ, and QZ contributed the software and other resources. YW and HX designed the study. HX and YLi revised the manuscript. All of the authors read and approved the final manuscript.

## Conflict of Interest

The authors declare that the research was conducted in the absence of any commercial or financial relationships that could be construed as a potential conflict of interest.
