# Supplementary material for: gga-miR-148a-5p-Targeting PDPK1 Inhibits Proliferation and Cell Cycle Progression of Avain Leukosis Virus Subgroup J (ALV-J)-Infected Cells
Source: Front Cell Dev Biol. 2020 Dec 15;8:587889. doi: 10.3389/fcell.2020.587889 (PMC7769946; doi:10.3389/fcell.2020.587889)
Supplement: Supplementary file 1 [file Table_1.DOCX]

Supplementary Material

Table S1.101 Genes in the NF-κB Pathway

| 101 Genes in the NF-kB Pathway | | | | | |
| --- | --- | --- | --- | --- | --- |
| [TRAF2](http://genecards.org/cgi-bin/carddisp.pl?gene=TRAF2) | [TRAF5](http://genecards.org/cgi-bin/carddisp.pl?gene=TRAF5) | [TICAM1](http://genecards.org/cgi-bin/carddisp.pl?gene=TICAM1) | [TRAM1](http://genecards.org/cgi-bin/carddisp.pl?gene=TRAM1) | [TRAM2](http://genecards.org/cgi-bin/carddisp.pl?gene=TRAM2) | [TRAM1L1](http://genecards.org/cgi-bin/carddisp.pl?gene=TRAM1L1) |
| [BCL10](http://genecards.org/cgi-bin/carddisp.pl?gene=BCL10) | [PRKCQ](http://genecards.org/cgi-bin/carddisp.pl?gene=PRKCQ) | [TRAF6](http://genecards.org/cgi-bin/carddisp.pl?gene=TRAF6) | [TAB1](http://genecards.org/cgi-bin/carddisp.pl?gene=TAB1) | [TAB2](http://genecards.org/cgi-bin/carddisp.pl?gene=TAB2) | [IKBKE](http://genecards.org/cgi-bin/carddisp.pl?gene=IKBKE) |
| [RELB](http://genecards.org/cgi-bin/carddisp.pl?gene=RELB) | [ATM](http://genecards.org/cgi-bin/carddisp.pl?gene=ATM) | [TNFAIP3](http://genecards.org/cgi-bin/carddisp.pl?gene=TNFAIP3) | [IRAK3](http://genecards.org/cgi-bin/carddisp.pl?gene=IRAK3) | [HDAC1](http://genecards.org/cgi-bin/carddisp.pl?gene=HDAC1) | [HDAC2](http://genecards.org/cgi-bin/carddisp.pl?gene=HDAC2) |
| [HDAC9](http://genecards.org/cgi-bin/carddisp.pl?gene=HDAC9) | [HDAC10](http://genecards.org/cgi-bin/carddisp.pl?gene=HDAC10) | [HDAC11](http://genecards.org/cgi-bin/carddisp.pl?gene=HDAC11) | [BTK](http://genecards.org/cgi-bin/carddisp.pl?gene=BTK) | [CD14](http://genecards.org/cgi-bin/carddisp.pl?gene=CD14) | [CD4](http://genecards.org/cgi-bin/carddisp.pl?gene=CD4) |
| [MAPK15](http://genecards.org/cgi-bin/carddisp.pl?gene=MAPK15) | [FADD](http://genecards.org/cgi-bin/carddisp.pl?gene=FADD) | [FYN](http://genecards.org/cgi-bin/carddisp.pl?gene=FYN) | [GBP2](http://genecards.org/cgi-bin/carddisp.pl?gene=GBP2) | [GBP1](http://genecards.org/cgi-bin/carddisp.pl?gene=GBP1) | [GBP5](http://genecards.org/cgi-bin/carddisp.pl?gene=GBP5) |
| [LCK](http://genecards.org/cgi-bin/carddisp.pl?gene=LCK) | [IRF6](http://genecards.org/cgi-bin/carddisp.pl?gene=IRF6) | [LYN](http://genecards.org/cgi-bin/carddisp.pl?gene=LYN) | [MALT1](http://genecards.org/cgi-bin/carddisp.pl?gene=MALT1) | [LY96](http://genecards.org/cgi-bin/carddisp.pl?gene=LY96) | [MAP3K3](http://genecards.org/cgi-bin/carddisp.pl?gene=MAP3K3) |
| [PIK3CB](http://genecards.org/cgi-bin/carddisp.pl?gene=PIK3CB) | [RIPK1](http://genecards.org/cgi-bin/carddisp.pl?gene=RIPK1) | [RIPK2](http://genecards.org/cgi-bin/carddisp.pl?gene=RIPK2) | [RPS6KA1](http://genecards.org/cgi-bin/carddisp.pl?gene=RPS6KA1) | [SUMO1](http://genecards.org/cgi-bin/carddisp.pl?gene=SUMO1) | [SUMO2](http://genecards.org/cgi-bin/carddisp.pl?gene=SUMO2) |
| [TLR4](http://genecards.org/cgi-bin/carddisp.pl?gene=TLR4) | [TNF](http://genecards.org/cgi-bin/carddisp.pl?gene=TNF) | [TNFRSF1A](http://genecards.org/cgi-bin/carddisp.pl?gene=TNFRSF1A) | [MAP3K8](http://genecards.org/cgi-bin/carddisp.pl?gene=MAP3K8) | [TRADD](http://genecards.org/cgi-bin/carddisp.pl?gene=TRADD) | [TRAF3](http://genecards.org/cgi-bin/carddisp.pl?gene=TRAF3) |
| [PRKCD](http://genecards.org/cgi-bin/carddisp.pl?gene=PRKCD) | [PRKCG](http://genecards.org/cgi-bin/carddisp.pl?gene=PRKCG) | [PRKCH](http://genecards.org/cgi-bin/carddisp.pl?gene=PRKCH) | [PRKCI](http://genecards.org/cgi-bin/carddisp.pl?gene=PRKCI) | [PRKCZ](http://genecards.org/cgi-bin/carddisp.pl?gene=PRKCZ) | CARD11 |
| [TIRAP](http://genecards.org/cgi-bin/carddisp.pl?gene=TIRAP) | [IRAK1](http://genecards.org/cgi-bin/carddisp.pl?gene=IRAK1) | [PLCG2](http://genecards.org/cgi-bin/carddisp.pl?gene=PLCG2) | [DAG1](http://genecards.org/cgi-bin/carddisp.pl?gene=DAG1) | [PRKCB](http://genecards.org/cgi-bin/carddisp.pl?gene=PRKCB) | TBK1 |
| [IKBKG](http://genecards.org/cgi-bin/carddisp.pl?gene=IKBKG) | [CYLD](http://genecards.org/cgi-bin/carddisp.pl?gene=CYLD) | [NFKB1](http://genecards.org/cgi-bin/carddisp.pl?gene=NFKB1) | [CHUK](http://genecards.org/cgi-bin/carddisp.pl?gene=CHUK) | [IKBKB](http://genecards.org/cgi-bin/carddisp.pl?gene=IKBKB) | PRKCA |
| [HDAC3](http://genecards.org/cgi-bin/carddisp.pl?gene=HDAC3) | [HDAC4](http://genecards.org/cgi-bin/carddisp.pl?gene=HDAC4) | [HDAC5](http://genecards.org/cgi-bin/carddisp.pl?gene=HDAC5) | [HDAC6](http://genecards.org/cgi-bin/carddisp.pl?gene=HDAC6) | [HDAC7](http://genecards.org/cgi-bin/carddisp.pl?gene=HDAC7) |  |
| [ECSIT](http://genecards.org/cgi-bin/carddisp.pl?gene=ECSIT) | [MAPK1](http://genecards.org/cgi-bin/carddisp.pl?gene=MAPK1) | [MAPK6](http://genecards.org/cgi-bin/carddisp.pl?gene=MAPK6) | [MAPK7](http://genecards.org/cgi-bin/carddisp.pl?gene=MAPK7) | [MAPK3](http://genecards.org/cgi-bin/carddisp.pl?gene=MAPK3) |  |
| [GBP6](http://genecards.org/cgi-bin/carddisp.pl?gene=GBP6) | [GSK3B](http://genecards.org/cgi-bin/carddisp.pl?gene=GSK3B) | [NFKBIA](http://genecards.org/cgi-bin/carddisp.pl?gene=NFKBIA) | [NFKBIB](http://genecards.org/cgi-bin/carddisp.pl?gene=NFKBIB) | [IRAK4](http://genecards.org/cgi-bin/carddisp.pl?gene=IRAK4) |  |
| [HLA-E](http://genecards.org/cgi-bin/carddisp.pl?gene=HLA-E) | [RPS6KA5](http://genecards.org/cgi-bin/carddisp.pl?gene=RPS6KA5) | [MYD88](http://genecards.org/cgi-bin/carddisp.pl?gene=MYD88) | [MAP3K14](http://genecards.org/cgi-bin/carddisp.pl?gene=MAP3K14) | [RELA](http://genecards.org/cgi-bin/carddisp.pl?gene=RELA) |  |
| [SUMO3](http://genecards.org/cgi-bin/carddisp.pl?gene=SUMO3) | [SUMO4](http://genecards.org/cgi-bin/carddisp.pl?gene=SUMO4) | [SYK](http://genecards.org/cgi-bin/carddisp.pl?gene=SYK) | [MAP3K7](http://genecards.org/cgi-bin/carddisp.pl?gene=MAP3K7) | [TANK](http://genecards.org/cgi-bin/carddisp.pl?gene=TANK) |  |
| [ZAP70](http://genecards.org/cgi-bin/carddisp.pl?gene=ZAP70) | [CD3E](http://genecards.org/cgi-bin/carddisp.pl?gene=CD3E) | [CD3G](http://genecards.org/cgi-bin/carddisp.pl?gene=CD3G) | [BCR](http://genecards.org/cgi-bin/carddisp.pl?gene=BCR) | [PRKCE](http://genecards.org/cgi-bin/carddisp.pl?gene=PRKCE) |  |
| NFKB2 | HDAC8 | MAPK12 | LBP | **PDK1** |  |
